# The Sheep as a Comprehensive Animal Model to Investigate Interdependent Physiological Pressure Propagation and Multiparameter Influence on Cerebrospinal Fluid Dynamics

**DOI:** 10.3389/fnins.2022.868567

**Published:** 2022-03-31

**Authors:** Nina Eva Trimmel, Anthony Podgoršak, Markus Florian Oertel, Simone Jucker, Margarete Arras, Marianne Schmid Daners, Miriam Weisskopf

**Affiliations:** ^1^Center for Surgical Research, University Hospital Zurich, University of Zurich, Zurich, Switzerland; ^2^Department of Mechanical and Process Engineering, ETH Zürich, Zurich, Switzerland; ^3^Department of Neurosurgery, University Hospital Zurich, University of Zurich, Zurich, Switzerland

**Keywords:** cerebrospinal fluid dynamics, intracranial pressure, translational neuroscience, ventriculoperitoneal shunt, hydrocephalus, intrathecal pressure, sheep model, craniospinal pressure

## Abstract

The present study aims to develop a suitable animal model for evaluating the physiological interactions between cerebrospinal fluid (CSF) dynamics, hemodynamics, and abdominal compartment pressures. We seek to contribute to the enhanced recognition of the pathophysiology of CSF-dependent neurological disorders like hydrocephalus and the improvement of available treatment options. To date, no comprehensive animal model of CSF dynamics exists, and establishing an accurate model will advance our understanding of complex CSF physiology. Persisting knowledge gaps surrounding the communication and pressure propagation between the cerebrospinal space and adjacent anatomical compartments exacerbate the development of novel therapies for neurological diseases. Hence, the need for further investigation of the interactions of vascular, craniospinal, and abdominal pressures remains beyond dispute. Moreover, the results of this animal study support the optimization of *in vitro* test benches for medical device development, e.g., ventriculoperitoneal shunts. Six female white alpine sheep were surgically equipped with pressure sensors to investigate the physiological values of intracranial, intrathecal, arterial, central venous, jugular venous, vesical pressure, and four differently located abdominal pressures. These values were measured simultaneously during the acute animal trial with sheep under general anesthesia. Both carotid and femoral arterial blood pressure indicate a reliable and comparable representation of the systematic blood pressure. However, the jugular venous pressure and the central venous pressure in sheep in dorsal recumbency do not correlate well under general anesthesia. Furthermore, there is a trend for possible comparability of lateral intraventricular and lumbar intrathecal pressure. Nevertheless, animal body position during measurements must be considered since different body constitutions can alter the horizontal line between the cerebral ventricles and the lumbar subarachnoid space. While intra-abdominal pressure measurement in the four different abdominal quadrants yielded greater inter-individual variability, intra-vesical pressure measurements in our setting delivered comparable values for all sheep. We established a novel and comprehensive ovine animal model to investigate interdependent physiologic pressure propagation and multiparameter influences on CSF dynamics. The results of this study will contribute to further *in vitro* bench testing, the derivation of novel quantitative models, and the development of a pathologic ovine hydrocephalus model.

## Introduction

Complete understanding of the complex physiology of cerebrospinal fluid (CSF) constitutes an integral component of understanding the pathophysiology of many neurological disorders and optimizing their treatment (Lyons and Meyer, 1990; Filis et al., 2017). One of the diseases originating from disturbed CSF dynamics is hydrocephalus, a complex neurologic condition with different classifications and pathophysiological causes. This condition typically causes the ventricles of the brain to expand in size, intracranial pressure (ICP) to rise, and CSF volume to increase (Mori et al., 1995). Hydrocephalus can generally occur in two clinical forms – congenital or acquired (Zhang et al., 2006). Various authors have further tried to classify hydrocephalus in detail, with the first being Dandy and Blackfan (1913), distinguishing between communicating and non-communicating hydrocephalus, and Russel (1949) describing obstructive and non-obstructive forms.

A type of hydrocephalus mainly affecting elderly patients is idiopathic normal pressure hydrocephalus (iNPH), first described by Hakim and Adams (1965) and Adams et al. (1965). In iNPH, a communicating form of hydrocephalus, a pathologic accumulation of CSF occurs in the ventricular system. However, in most cases of iNPH, ICP is not increased. This lack of pressure build-up in the ventricles exacerbates standard shunt-based medical treatment by hindering proper functionality of existing pressure-dependent valves (Graff-Radford and Jones, 2019; Lalou et al., 2021). Nevertheless, iNPH is usually treated by surgically implanting a shunt system with a one-way differential pressure valve that either opens at a fixed pressure or can be adjusted externally to open at a predetermined pressure level (Sæhle et al., 2014). Today’s most ubiquitous system is the ventriculoperitoneal (VP) shunt, which connects the ventricles of the brain with the intraperitoneal cavity, thereby providing a pathway to drain excessive CSF (Reddy et al., 2014). However, the complication rate in VP shunt treatments for iNPH patients remains high, with 42.5% encountering over- or under-drainage (Hung et al., 2017) requiring frequent revisions that can lead to poor treatment outcomes (Kestle et al., 2000; Rocque et al., 2021). Nevertheless, since iNPH affects mainly the elderly, the persistence of cardinal symptoms (e.g., gait disturbance, urinary incontinence, and cognitive impairment) after treatment may also have originated in different comorbidities, such as dementia, Alzheimer’s disease, or other neurological conditions (Graff-Radford and Jones, 2019).

Other types of shunts currently exist, e.g., lumboperitoneal, ventriculopleural, or ventriculoatrial (VA) shunts, each named after their respective drainage location (Giordan et al., 2019). Ventriculopleural shunts and lumboperitoneal shunts are not widely used to treat hydrocephalus but are occasionally considered a third-line treatment option if the use of a VP or VA shunt is contraindicated (Baro et al., 2020; Todisco et al., 2020). In the early days of shunt treatments, VP shunts yielded higher morbidity rates; thus, VA shunts were the primary choice (Rymarczuk et al., 2020). However, VP shunts are now associated with lower complication rates, specifically with iNPH patients (Hung et al., 2017; Rymarczuk et al., 2020). Nevertheless, since VP shunts work with a pressure-dependent valve system, they often fail to function sufficiently in iNPH patients.

Moreover, conventional shunt systems fail to adequately address the need for slow CSF drainage in iNPH patients to prevent rapid functionality changes, compared with patients with other forms of hydrocephalus such as children, where fast CSF removal is needed to ensure normal brain development (Reddy et al., 2014; Sæhle et al., 2014; Filis et al., 2017). Hence, there is a need for intensified research into improved shunt systems to develop a solution for iNPH patients and ultimately create shunt systems that do not rely solely on pressure-dependent valves to function. Such shunt-systems would need to take other parameters, e.g., CSF volume, into account to target the iNPH-specific pathology and actuate CSF drainage.

Postural state, arterial blood pressure (ABP), as well as venous and lymphatic drainage, play an essential role in maintaining physiological fluid dynamics and pressure balance due to their influence on cerebral blood flow and cerebral perfusion pressure (CPP) (Sakka et al., 2016; Czosnyka et al., 2019; Ma et al., 2019). The interaction and interdependence of the various compartments were previously shown in a porcine model demonstrating the effect of an increase in intra-abdominal pressure (IAP) with and without postural changes on ICP, mediated by altered central venous pressure (CVP) due to mechanical constriction of the inferior vena cava (Rosenthal et al., 1998). The elevated ICP was hypothesized to cause a subsequent increase in mean arterial pressure (MAP) to maintain physiological perfusion pressure in the brain (Rosenthal et al., 1998). However, it has been shown that location-dependent gravitational and non-gravitational pressure gradients, caused by diaphragmatic contraction or simple displacement of abdominal content, can be found within the abdominal cavity (Loring et al., 1994; Hurcombe and Scott, 2012; De Paula et al., 2019), raising the question of whether pressure changes in some specific regions within the peritoneal cavity may have less effect on adjacent compartments than others.

Currently, the design of VP shunt systems does not adequately account for these dynamic pressure changes in the abdominal cavity. Intra-abdominal hypertension at the location of catheter placement could hinder CSF flow from the ventricles to the peritoneal space, causing shunt malfunction *via* occlusion (Bloomfield et al., 1997; Sato et al., 2020).

The need for further investigation into the dynamics and interactions of shunts, valves, the ventricular system, and CSF properties remains beyond dispute (Bergsneider et al., 2008). However, developing an improved drainage system is currently prevented by unclear technical feasibility, questionable economic return, and high regulatory burdens (Lutz et al., 2013). Furthermore, a lack of suitable large animal models for pre-clinical testing that would give a comprehensive understanding of dynamic pressure and volume variations and interdependencies in different body compartments exists today. Cats, dogs, and pigs have been used widely in the past to study VP function and ICP pressure dynamics (Rosenthal et al., 1998; Bayston et al., 2008; Klarica et al., 2009; Iyer et al., 2018; Qin et al., 2019). A limiting factor when using cats for hydrocephalus research is their relatively small total CSF volume of 1,300 μL compared to 150 mL in humans (Levinger and Edery, 1968; Sakka et al., 2011), which could diminish the translational value of experiments. Cats also have a greater tendency for stronger stress responses and yield quite complicated species-specific behavior, making the housing of cats more demanding and costly (Geret et al., 2011). Although dogs remain valuable models for biomedical research due to their appropriate size, and their similarities in anatomy and physiology to humans allowing for therapeutic interventions studied in dogs to be translated rapidly into human clinical trials, a trend toward replacing dogs in biomedical research is underway. Public perception and financial motives with feline and canine studies being expensive to conduct will continue to reduce the number of dogs and cats used in biomedical research in the future (National Research Council [US] Committee on Scientific and Humane Issues in the Use of Random Source Dogs and Cats in Research, 2009; Hasiwa et al., 2011).

The porcine brain is similar to the human brain with respect to anatomy, growth, development, and biochemistry, leading to increased use of pig brains in neuroscience research (Lind et al., 2007). However, the presence of a large frontal sinus and the oblique auditory canals, as well as inter-individual variation in brain size and external skull structures of available pig breeds, may complicate standardized lateral ventricle targeting. Furthermore, pigs are unsuitable for long-term studies due to their fast somatic growth and rough behavior, which could cause displacement or even damage implants (Van Eerdenburg and Dierx, 2002).

Sheep have also previously been used to study hydrocephalus and CSF physiology (Di Rocco et al., 1977, 1978; Cambria et al., 1984; Di Trapani et al., 1990; De Keersmaecker et al., 2005; Johnston et al., 2013; Duru et al., 2019; Oria et al., 2019; Emery et al., 2020). However, little can be found in current literature describing physiologic values for the CSF space and the adjacent compartments in sheep. Therefore, the purpose of our study was to establish a comprehensive sheep model with a multitude of simultaneous pressure measurements in the CSF space and adjacent compartments, namely the intra-abdominal cavity and the vascular compartments. Studies of interdependent mechanisms of CSF pressure dynamics will foster the understanding of the physiological mechanisms needed to improve medical devices such as VP shunts. The acute physiological model presented in this paper is the foundation for further evaluation of interactions and interdependencies of the various compartments in long-term awake healthy sheep and pathological models.

## Materials and Methods

### Ethics Statement

Animal housing and all experimental procedures were approved by the local Committee for Experimental Animal Research (Cantonal Veterinary Office Zurich, Switzerland) under the license number ZH119/2019 in conformity with the European Directive 2010/63/EU of the European Parliament and the Council on the Protection of Animals used for Scientific Purposes, and the Guide for the Care and Use of Laboratory Animals procedure (National Research Council [US] Committee for the Update of the Guide for the Care and Use of Laboratory Animals, 2011).

### Animals

Six female white alpine sheep (*Ovis gmelini* aries) were included in this acute *in vivo* trial. All sheep were mature (2–5 years of age) and had a mean body weight of 75.6 ± 12.1 kg. After arrival, the sheep were housed in groups, with at least two animals in one pen, and allowed an adaptation period to the new environment of at least 1 week. The health status of the animals was guaranteed by the conventional livestock supplier through regular screening and subsequent official attestation according to the guidelines of the Swiss Federal Food Safety and Veterinary Office (FSVO) and determined *via* clinical examination by an on-site veterinarian. The animals had *ad libitum* access to water and hay. A salt licking stone was provided for all sheep. Room temperature was kept at a constant 19°C with a relative humidity of 45–55%. Experiments within the scope of this study were performed during the winter months in the northern hemisphere. Lights were on in all animal rooms from 7:00 am to 5:00 pm, and each room in the facility was equipped with an additional skylight to provide adequate daylight of approximately 10 h. The general condition of each animal was checked twice daily by the animal caretakers and regularly by a veterinarian.

### Preparation and Anesthesia

One day before surgery, sheep were deprived of food for approximately 16–18 h. They received water *ad libitum* during the fasting period. On the day of surgery, an intravenous catheter (Braunüle^®^MT Luer Lock, 14 G, B. Braun Medical, Melsungen, Germany) was placed in the jugular vein. The animal was then pre-medicated with 3 mg/kg BM ketamine hydrochloride (Ketasol^®^100, Dr. E. Graeub AG, Bern, Switzerland) and 0.3 mg/kg BM midazolam (Dormicum^®^ Roche Pharma, Reinach, Switzerland) intravenously. The animal was placed in a sternal position, and, prior to orotracheal intubation, anesthesia was induced with 2–5 mg/kg BM of propofol (Propofol-^®^Lipuro 1%, B. Braun Medical AG, Sempach, Switzerland) intravenously. A double-lumen urinary catheter (Rüsch Gold 3way, 18 F, Teleflex Medical Enterprise, Athlone, Ireland) was inserted into the bladder under sterile conditions. The double-lumen catheter provided another port for continuous intravesical pressure (IVP) measurements. An orogastric tube (Rüsch 12 mm, Teleflex Medical Enterprise, Athlone, Ireland) was placed to drain excess gastric fluid and relieve ruminal gases. A naso-esophageal temperature probe was inserted through the ventral nasal meatus for continuous body temperature monitoring. Anesthesia was maintained throughout the experiment by inhalation of 1–2% isoflurane (Attane*™* Isoflrane ad.us.vet., Piramal Enterpr. India, Lyssach, Switzerland) (fresh gas flow 1–1.5 L/min, 18 breaths/min, tidal volume 10–15 mL/kg BM, FiO2 0.7, Pmax 30 mmHg) in an oxygen/air mixture *via* volume-controlled ventilation through a semi-closed breathing circuit (Dräger Primus^®^, Dräger Medical, Lübeck, Germany). Anesthesia was balanced with a constant rate infusion of propofol (2–4 mg/kg/h). Intraoperative analgesia was provided by a constant rate infusion of sufentanil (Sufenta^®^forte, Janssen-Cilag AG, Zug, Switzerland; 2.5 μg/kg BM/h, i.v.). Ringer solution (Ringerfundin^®^, B. Braun Medical AG, Sempach, Switzerland) was administered intravenously at an infusion rate of 5 mL/kg/h.

### Instrumentation

**FIGURE 1:**
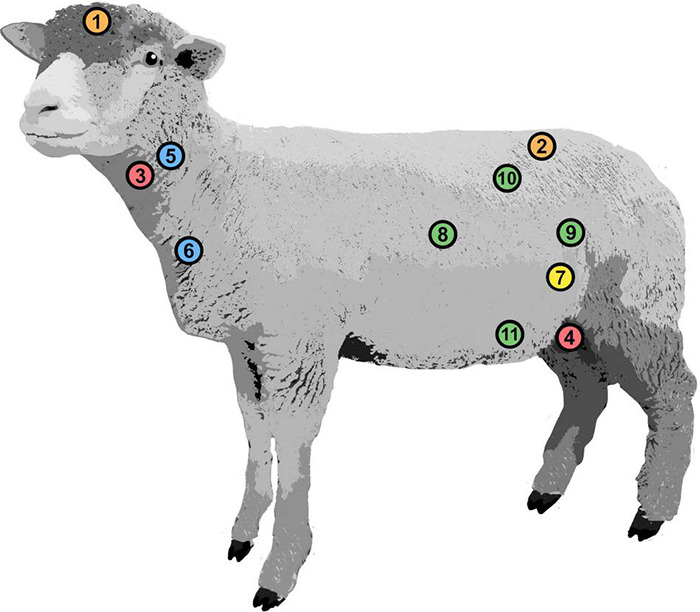
Positions of implanted pressure probes. Intracranial pressure (1), intrathecal pressure (2), carotid arterial pressure (3), femoral arterial pressure (4), jugular venous pressure (5), central venous pressure (6), intravesical pressure (7), intra-abdominal pressure: cranial (8), caudal (9), dorsal (10), and ventral (11).

Corresponding pressures were measured at 11 body locations by inserting catheters endovascularly or surgically in their respective positions (Figure 1). After implantation, the catheters were connected to a pressure acquisition device.

#### Placement of Intravascular Catheters for Pressure Measurements

**FIGURE 2:**
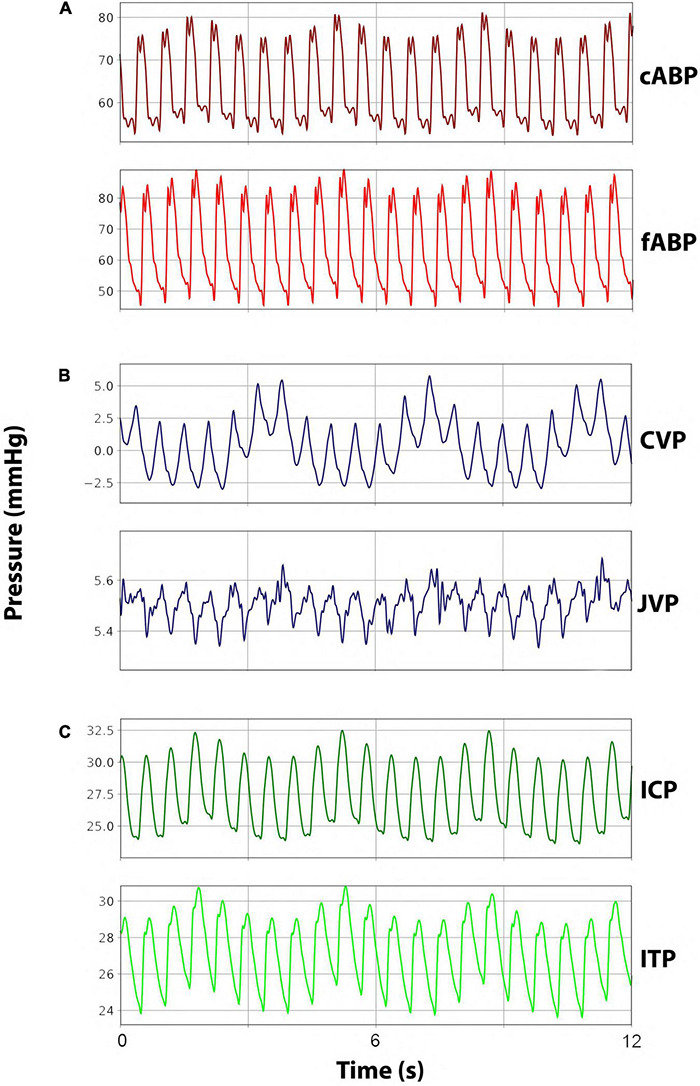
Representative pressure waveforms. **(A)** Arterial blood pressure curves for carotid arterial blood pressure (cABP) and femoral arterial blood pressure (fABP). **(B)** Venous blood pressure curves for jugular venous pressure (JVP) and central venous pressure (CVP). **(C)** Cerebrospinal fluid pressure curves for intracranial pressure (ICP) and intrathecal pressure (ITP).

Intravascular catheters were placed under sonographic guidance (iE33, Philips, Amsterdam, Netherlands) with the Seldinger method for continuous measurement of carotid arterial blood pressure (cABP), femoral arterial blood pressure (fABP), jugular venous pressure (JVP), and CVP. A 10 F arterial sheath (Avanti^®^, Cordis^®^Corporation, Miami Lakes, FL, United States) was placed in the femoral artery for fABP measurements. The carotid artery was catheterized with a 4 F introducer sheath (Avanti^®^, Cordis^®^Corporation, Miami Lakes, FL, United States) for cABP measurement. The single-lumen catheter in the jugular vein was exchanged for a triple-lumen catheter (Arrow^®^Multilumen Central Venous Catheterization Set with Blue Flextip^®^ Catheter, Arrow Int. Inc., Reading, PA, United States) to measure CVP as well as administer fluids, anesthetics, and medication. Another 4 F catheter was placed cranioproximal from the jugular sheath in the jugular vein to measure JVP. Representative arterial and venous pressure curves can be seen in Figures 2A,B.

#### Implantation of Intraventricular Pressure Probes

A small frontal burr hole trephination was performed using a diamond drill. Subsequently, two differently sized catheters, one 9 F external intraventricular catheter (Neuromedex GmbH, Hamburg, Germany) and one 4.5 F intrathecal catheter (Neuromedex GmbH, Hamburg, Germany), were inserted into the right lateral ventricle to measure real-time ICP and perform intraventricular volume infusions and extractions. A bone wax plug (Ethicon^®^ Bone wax, Johnson & Johnson Medical Ltd., Livingston, United Kingdom) was molded around both exiting catheters to plug the burr hole in the cranium, thus ensuring proper fixation and avoiding CSF leakage. Lastly, the 4.5 F catheter was connected to the pressure transducer, and the 9 F catheter was closed adequately with a stop cap before use. Representative ICP curves can be seen in Figure 2C.

#### Implantation of Intrathecal Pressure Probes

Before the surgical procedure, an X-ray of the lumbar spine was recorded (Allura Xper FD 20, Philips Medical Systems Netherlands BV, Best, Netherlands). The interlaminar window between L6 and L7 was marked. A 16 G epidural Tuohy cannula (Perifix^®^ 310 Mini Set, 16 G, B.Braun Melsungen AG, Melsungen, Germany) for CSF extractions and infusions and a 4.5 F intrathecal catheter (Neuromedex GmbH, Hamburg, Germany) for real-time intrathecal pressure (ITP) measurements were placed in the subarachnoid space *via* a laminotomy and minimal dural incision. The 4.5 F intrathecal catheter was advanced cranially into the intrathecal space by the aid of a dissector along the dorsal aspect of the spinal cord. The epidural cannula was inserted caudally and placed within the subarachnoid space as distal as surgically possible. A hemostatic gauze strip (Tabotamp^®^ Ethicon, Johnson & Johnson Medical, Neuchatel, Switzerland) was placed over the opening in the dura to avoid relevant CSF loss and blood entering the intrathecal space. Bone wax was molded around the exiting catheter to plug the bony defect, ensure proper catheter fixation, and prevent CSF leakage. After the surgical procedure, the intrathecal catheter was connected to the pressure transducers. Representative ITP curves can be seen in Figure 2C.

#### Implantation of Intra-Abdominal Pressure Probes

**FIGURE 3:**
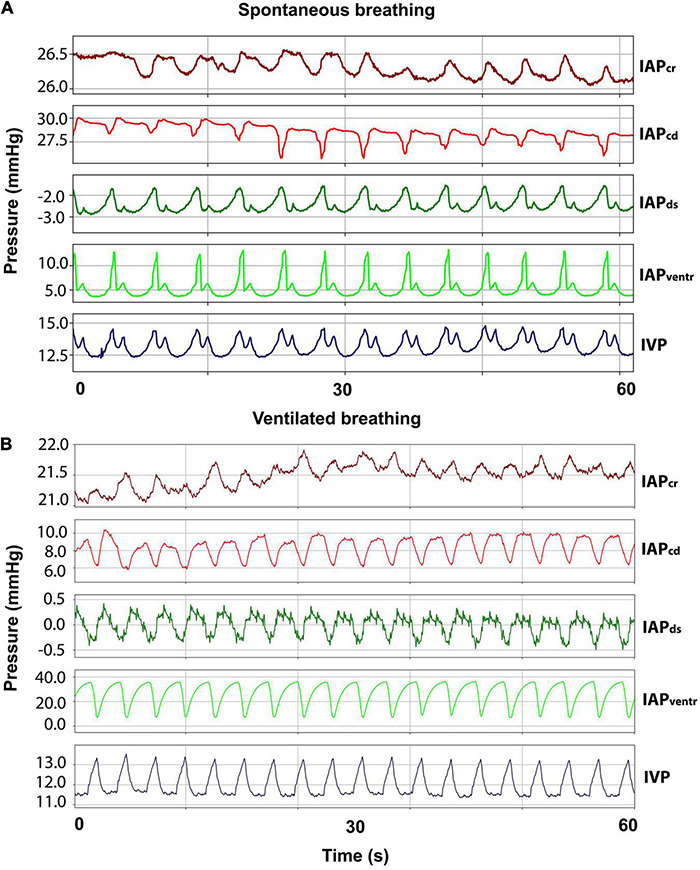
Respiratory abdominal waveforms. **(A)** Curves for cranial (IAP_cr_), caudal (IAP_cd_), dorsal (IAP_ds_), and ventral (IAP_ventr_) intraabdominal pressure as well as intravesical pressure (IVP) during spontaneous breathing. **(B)** IAP and IVP curves during ventilated breathing.

An incision of approximately 3–5 cm was made bilaterally on the flanks of the sheep. After dissection of the subcutis and muscle layers, the peritoneum was opened carefully. In each opening, two 10 F access sheaths (Avanti^®^, Cordis^®^ Corporation, Arrow Int. Inc., Reading, PA, United States) were inserted and positioned in each of the four different quadrants of the abdominal cavity. Catheters for cranial IAP (IAP_cr_) and caudal IAP (IAP_cd_) were inserted opposite each another into the right abdominal wall. Ventral IAP (IAP_ventr_) and dorsal IAP (IAP_ds_) were measured by inserting the catheters into the left abdominal wall in an opposing manner. Abdominal integrity was restored by closing the peritoneum and fascia continuously and the skin by making single-knots and purse-string sutures around the inserted catheters. Finally, the pressure transducers were fixated to the sheep’s skin *via* Backhaus clamps after connecting them to the catheters. Abdominal pressures were measured during ventilated and spontaneous breathing. Representative curves for abdominal pressures during spontaneous and ventilated breathing can be seen in Figures 3A,B.

### Pressure Data Acquisition

Intraventricular and intrathecal transducers were zeroed to atmospheric pressure at the level of the lateral ventricles to obtain hydrostatic equivalence between both. The arterial and central venous sensors were zeroed at the right atrium level, and the jugular venous sensor was zeroed at the tip of the jugular pressure probe. The sheep remained in sternal recumbence throughout the experiment. This posture corresponds to the horizontal position in humans, where ITP and ICP are assumed to be unaffected by hydrostatic fluctuations. IVP was measured *via* a two-lumen urinary Foley catheter, with one opening connected to the pressure transducer and the second opening serving as a urinary outlet. Urine was removed from the bladder before each intervention. After urine removal, a standard volume of 20 mL saline solution was infused into the bladder *via* the catheter to standardize IVP measurement.

Pressure data were acquired during an undisturbed 10-min baseline, using the commercially available software Ponemah v5.1 (Data Science International, ST. Paul, MN, United States) combined with the ACQ-7700 acquisition unit. To amplify the signal, the Universal XE and ABCD 4 were used. The catheters were connected to DTXPlus pressure transducers (Argon Medical Devices, The Hague, Netherlands). Data acquisition was performed at a sampling frequency of 1 kHz, then discriminated to 100 Hz and analyzed using custom scripts written in Python 3.7.10 (Open Source, Python Software Foundation, Wilmington, DE, United States). Baseline mean pressures and pulse amplitudes were calculated as 5-min arithmetic means, and values are described as mean ± SD. For each sheep, the 10-min cABP baseline data was separated into its respective frequency components *via* Fast Fourier Transform (FFT), followed by the separation of the cardiac band. The heart rate was then calculated individually for each sheep as the peak of the cardiac band within the FFT spectrum. Finally, the arithmetic means and standard deviation between sheep were calculated in Microsoft Excel (Microsoft Corporation, Redmond, WA, United States).

### Euthanasia

At the end of the experiment, sheep were humanely euthanized under deep general anesthesia by administering pentobarbital (Eskonarkon^®^ad.us.vet., Streubli Pharma AG, Uznach, Switzerland) (75 mg/kg BM) intravenously.

## Results

### Intracranial Pressure and Intrathecal Pressure

Table 1 lists the pressures for each sheep. The average mean ICP for all sheep was 13.9 ± 9.4 mmHg, and the average mean ITP was 10.6 ± 12.0 mmHg. Sheep B and F had an ICP and ITP above 20 mmHg during baseline measurements.

**TABLE 1:** Mean cerebrospinal fluid pressures measured during a 10-min baseline.

Sheep	A	B	C	D	E	F
ICP (mmHg)	2.2 ± 0.1	20.7 ± 0.8	6.5 ± 0.2	10.0 ± 0.0	16.7 ± 0.0	27.2 ± 0.3
ITP (mmHg)	0.4 ± 0.2	23.9 ± 1.2	6.2 ± 0.9	0 ± 0.3	5.6 ± 4.5	27.2 ± 0.2

*Mean intracranial pressure (ICP) and mean intrathecal pressure (ITP) for each sheep, measured during a baseline of 10 min.*

Sheep A and D had ITP values leveling around 0 mmHg but displayed physiologic ICP levels in a range of 2.2 and 10 mmHg. Sheep C showed comparable ICP and ITP values in a physiologic range of 6.5 and 6.2 mmHg. Sheep E had a greater difference between ICP with 16.7 mmHg and ITP of only 5.6 mmHg. The ITP in sheep E showed the most marked fluctuation, as evidenced by the relatively high standard deviation of 4.6 mmHg compared to the other sheep.

### Hemodynamics

#### Mean Carotid Arterial Pressure and Mean Femoral Arterial Pressure

Due to protocol adjustments, no mean femoral arterial blood pressure (mfABP) was acquired in sheep A and B. Sheep C, and F showed lower mean carotid arterial blood pressures (mcABP) and mfABP compared with the other sheep. For a complete list of the arterial pressures, see Table 2. The average mcABP for all sheep was 80.1 ± 11.9 mmHg, and the average mfABP (four sheep) was 75.4 ± 10.7 mmHg.

**TABLE 2:** Mean hemodynamic pressures and heart rate measured during a 10-min baseline.

Sheep	A	B	C	D	E	F
mcABP (mmHg)	84.6 ± 1.5	91.8 ± 1.0	65.9 ± 0.7	83.8 ± 2.8	89.8 ± 0.4	64.4 ± 0.7
mfABP (mmHg)	n.a.	n.a.	67.6 ± 11.3	81.1 ± 8.6	87.5 ± 5.6	65.2 ± 12.6
Heartrate (bpm)	93.8 ± 0.0	119.4 ± 0.0	111.8 ± 0.0	96.1 ± 0.0	104.2 ± 0.0	103.5 ± 0.0
JVP (mmHg)	n.a.	n.a.	-3.9 ± 1.9	7.8 ± 0.7	5.7 ± 0.1	7.3 ± 0.4
CVP (mmHg)	4.6 ± 0.1	5.3 ± 0.2	4.7 ± 0.9	2.9 ± 0.3	1.3 ± 0.0	3.9 ± 0.1

*Mean carotid arterial blood pressure (mcABP), mean femoral arterial blood pressure (mfABP) (except for sheep A and B), mean heart rate in beats per minute (bpm) as well as mean jugular venous pressure (JVP) (except for sheep A and B) and central venous pressure (CVP) for each sheep, measured during a baseline of 10 min.*

#### Heart Rate

The heart rates of all sheep during baseline measurements presented similar values. The average mean heart rate for all sheep was 97.8 ± 13 bpm. For a complete list of sheep heart rates, see Table 2.

#### Jugular Venous Pressure and Central Venous Pressure

Due to experimental protocol changes, no JVP data are available for sheep A, and B. Sheep D and F had elevated JVP values compared with the other sheep. Sheep C showed a negative JVP during baseline measurements but had a positive CVP value. The average mean JVP for all sheep was 4.2 ± 5.5 mmHg, and the average mean CVP was 3.8 ± 01.5 mmHg. See Table 2 for a complete list of all venous pressures.

### Abdominal Pressures and Intravesical Pressure

A complete list of abdominal pressures of each sheep can be seen in Table 3. The mean IVP for all sheep was 12.2 ± 2.2 mmHg. The mean IAP_cr_ was 13.5 ± 11.8 mmHg, whereas the IAP_cd_ was 11.7 ± 14.2. For the IAP_ds_, the mean was calculated at 12.9 ± 14.2, and the mean IAP_ventr_ was calculated at 11.7 ± 11.5. IVP measurements presented comparable values for all sheep, with the lowest IVP at 9.4 ± 9.5 mmHg and the highest at 15.6 ± 8.1 mmHg. The IAP_cr_ showed higher pressure value variability than the IAP_cd_, where only sheep F had an average increased IAP_cd_ of 40.4 ± 3.4 mmHg. The IAP_ds_ behaved similarly to the IAP_cd_, whereas the IAP_ventr_ does not show consistency.

**TABLE 3:** Mean intra-abdominal pressures measured during a 10-min baseline.

Sheep	A	B	C	D	E	F
Bladder (mmHg)	10.9 ± 5.4	13.6 ± 2.6	9.4 ± 9.5	12.3 ± 0.6	15.6 ± 8.1	11.3 ± 0.8
IAP_cr_ (mmHg)	32.7 ± 2.1	6.2 ± 2.1	19.3 ± 15.7	17.0 ± 4.5	0.92 ± 2.2	5.0 ± 8.3
IAP_cd_ (mmHg)	5.3 ± 1.3	8.5 ± 1.9	3.1 ± 2.8	7.5 ± 0.8	5.4 ± 3.9	40.4 ± 3.4
IAP_ds_ (mmHg)	5.4 ± 1.5	7.9 ± 8.1	13.9 ± 9.1	-2.3 ± 0.2	13.2 ± 8.2	39.2 ± 0.4
IAP_ventr_ (mmHg)	23.2 ± 5.6	6.3 ± 0.6	6.6 ± 1.8	29.1 ± 13.2	4.2 ± 1.0	0.9 ± 0.5

*Mean intra-abdominal pressures in each intraperitoneal quadrant cranial (IAP_cr_), caudal (IAP_cd_), dorsal (IAP_ds_), and ventral (IAP_ventr_) as well as the intravesical pressure (IVP) for each sheep, measured during a baseline of 10 min.*

### Pressure Correlations

**FIGURE 4:**
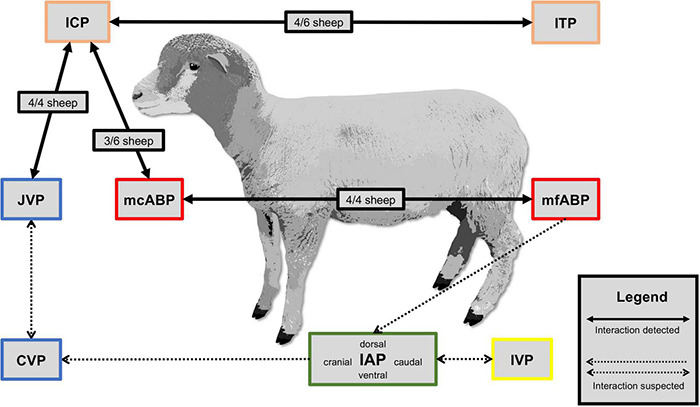
Pressure correlation. Visualization of detected and suspected body compartment interactions between intracranial pressure (ICP), intrathecal pressure (ITP), mean carotid arterial pressure (mcABP), mean femoral arterial pressure (mfABP), jugular venous pressure (JVP), central venous pressure (CVP), intravesical pressure (IVP), and intra-abdominal pressure (IAP) measured during a baseline of 10 min.

In 3 out of 6 sheep, a baseline measurement correlation between ICP and ITP was found and, during the subsequent perfusion tests, communication existed in 4 out of 6 sheep. ICP and ITP did not correlate in Sheep A, D, and E during baseline measurements, but Sheep D displayed communication between ICP and ITP during tap and infusion tests. In 3 out of 6 sheep, ICP was high when arterial blood pressure was low, compared with the remaining animals. When comparing mcABP and mfABP, it was found that both pressures agreed in all of the four sheep in which these pressures were measured (Sheep C, D, E, and F). In all four sheep, in which JVP was measured (Sheep C, D, E, and F), JVP displayed higher pressure levels where there was increased ICP. ICP was higher in sheep B, D, and E and, in these three sheep also displayed a higher ABP compared with the other sheep. In Sheep F, where ICP was markedly more elevated with 27.2 mmHg, ABP remained lower (64.4 mmHg), although an increased JVP of 7.3 mmHg was observed. During the baseline measurements, no correlation between IVP and IAP in any of the four quadrants was found. The same conclusion was drawn when comparing abdominal pressures with all hemodynamic parameters and CSF pressures. Figure 4 presents a visualization of the pressure correlation results.

## Discussion

While current research on hydrocephalus focuses on the general area of biology (Asgari et al., 2015) and biological processes associated with CSF circulation (Brinker et al., 2014; Krishnamurthy et al., 2018; Hladky and Barrand, 2019), establishing an animal model investigating intercompartmental pressure dynamics will bridge the knowledge gap on pathophysiologic intracranial dynamics and their interdependencies with other body compartments.

### Intracranial Pressure and Intrathecal Pressure

In humans, craniospinal pressure is commonly measured while the subject is lying in a horizontal recumbent position. The pressure is assumed to be the same along the spinal subarachnoid space and inside the cranium (Davson et al., 1987; Lenfeldt et al., 2007). Taking a simplified view, it is assumed that the pressure gradients between the intracranial and along the subarachnoid space are formed based solely on hydrostatic differences. Although this simplistic mechanism of regulating craniospinal pressure gradients has been questioned, at least in horizontally positioned cats, a uniform CSF pressure behavior has proven true (Klarica et al., 2014). We aimed for a comparable baseline situation by placing all sheep in a sternal position. However, the different body constitutions of each individual sheep may have altered the horizontal line between the cerebral ventricle and the lumbar subarachnoid space to a rather convex shape, causing posture-dependent hydrostatic pressure gradients. Although the data were corrected for this hydrostatic pressure, ICP and ITP pressure were aligned in only 3/6 sheep (B, C, and F), while in the other 3 animals (A, D, and E), ITP was markedly lower than ICP. Volume infusions and extractions performed within the same series of experiments showed non-communication between the ITP in the subarachnoid space and the intracranial CSF space in sheep A and E (Podgoršak et al., 2022). Considering the simplified bulk flow theory, CSF is thought to be produced by the choroid plexus throughout the ventricular system, from where it flows through the interventricular foramina of Monro into the third ventricle in the midbrain, before continuing through the cerebral aqueduct, to the fourth ventricle. Afterward, it exits through the foramina of Luschka and Magendie into the central spinal canal and subarachnoid space (Brodbelt and Stoodley, 2007; Brinker et al., 2014; Yamada, 2014). One can only speculate about the underlying causes of non-communication between the craniospinal space and the right lateral ventricle, where our ICP pressure probe was placed. In the non-communicating sheep, the intracranial baroreflex, described by Vari et al. (2021), could be triggered by intrathecal volume infusion, as shown recently by our research group by performing infusion and extraction tests in this present sheep model (Podgoršak et al., 2022). It might be assumed that the induced change in volume and pressure still reached the intracranial space, but not the right lateral ventricle.

Previous studies reported 4.0 ± 1.1 mmHg (Guild et al., 2018), 6.4 ± 1.9 mmHg (Wells et al., 2015), and 7.0 ± 1.0 mmHg (Vari et al., 2021), respectively, as physiological ICP values in sheep. While 4/6 sheep in this study had ICP levels following normal physiologic values, 2/6 (Sheep B and F) had an ICP value > 20 mmHg, which is considered pathologic (Jantzen, 2007). An increased ICP in sheep can have a variety of underlying causes, such as traumatic brain injury (Vink et al., 2008), ischemic stroke (Wells et al., 2015), or infection (Finnie, 2003). Furthermore, anesthesia-related causes such as hypoxia (Iwamoto et al., 1991) or hypercapnia (Cassaglia et al., 2008), anesthetic agents such as isoflurane or desflurane (Artru et al., 1994; Brosnan et al., 2002), and animal positioning (Brosnan et al., 2002) are known contributors to intracranial hypertension (ICH). As ventilation was controlled in all sheep in the present study, hypoxia or hypercapnia can be widely excluded as a cause for increased ICP. The decreased mean ABP measured in Sheep F might indicate a deeper anesthetic plane, possibly induced by increased isoflurane concentrations. A low mean ABP combined with an increased ICP can reduce CPP, thus leading to the assumption that cerebral autoregulation was hampered by a deep anesthetic plane in this sheep (Dagal and Lam, 2009). Another plausible explanation for ICH in Sheep F might be the increased IAP measured in the caudal and dorsal quadrant. An increase in IAP has been shown to lead to a cranial displacement of the diaphragm, increased intrathoracic pressure, and compression of the vena cava, which in turn will increase sagittal sinus pressure and ICP (Rosenthal et al., 1998). As sagittal sinus pressure was not assessed, and JVP is thought to increase only at ICP levels of > 40 mmHg (Johnston and Rowan, 1974), no conclusive statement can be made at this point. Sheep B, on the other hand, showed an increased MAP and an increased heart rate, indicating an autoregulatory response to an increased ICP to sustain CPP. All IAPs were in a physiological range between 0 and 13 mmHg (Hartman, 1973) in sheep B. Therefore, the elevation in ICP is suspected of having been induced by catheter placement or an injury to the cribriform plate through the naso-esophageal insertion of the temperature probe. Disturbance of the lymphatic drainage at the cribriform plate reduces CSF drainage in sheep (Silver et al., 2002) and consequently increases ICP.

### Hemodynamics

Jugular venous pressure is not commonly measured during clinical interventions in humans because there is no exact correlation with the right atrial pressure of the heart and it is, furthermore, often mismeasured. However, some authors believe that JVP measurement is less invasive than CVP measurements and yields fewer complications (Abdullah et al., 2011; Chua Chiaco et al., 2013). According to clinical studies performed in humans, there is no significant pressure difference between the external JVP and the CVP in the supine position (Parker et al., 2002; Leonard et al., 2008). As seen in humans during the perpendicular posture, the collapse of the external jugular vein acts as a preventive mechanism by constricting blood flow from the cranium to the heart. This mechanism maintains the CPP and ensures a stable ICP (Holmlund et al., 2018). This collapse can lead, in turn, to a difference between JVP and CVP. To our knowledge, in sheep, JVP and the total collapse of the common jugular vein in an upright position have not yet been investigated. These data would have to be addressed in a future study.

In horses, it was found that CVP correlated well with JVP in the lateral recumbency (Tam et al., 2011), whereas one study in dogs and cats found a poor correlation between JVP and CVP measurements (Chow et al., 2006). In our present study, one animal had a negative JVP, likely associated with a blockage in the vascular sheath or valves lying proximal to the pressure probe tip. Elevations in intrathoracic pressure also influence the variability between CVP and JVP and can vary considerably during spontaneous breathing (Leonard et al., 2008). In 3/4 animals, JVP was higher than CVP. Since our sheep were ventilated *via* volume-controlled ventilation during baseline measurements, we suspect that ventilation caused the high variability in JVP and CVP measurements. In the present study, JVP was measured to get as close as possible to sagittal sinus pressure levels, which we have not measured directly.

Nevertheless, as JVP measurements were not reliable in this setting, since both catheters were placed in the same jugular vein, no conclusions regarding sagittal sinus pressure can be drawn. Interference between both pressure probes cannot be excluded.

The two pressures that behaved similarly throughout the baseline measurements in all sheep, were both mcABP and mfABP. Even in sheep with elevated intra-abdominal pressures, comparable mcABP and mfABP following previous experiments in pigs were found (Aksakal et al., 2012). We therefore conclude that both locations can be used for systemic ABP measurements equally if the surgical setting or study design allows it.

### Abdominal Pressure and Intravesical Pressure

The IAP is an essential, and often underestimated or neglected, factor for optimal VP shunt function (Sahuquillo et al., 2008). The indirect measurement of IAP *via* the bladder is still the standard method in humans and animals (Lee et al., 2002; Al-Abassi et al., 2018), completely neglecting gravitational and shear-associated abdominal pressure gradients (Loring et al., 1994; Hurcombe and Scott, 2012). Aside from a gravitational gradient directly affected by body positioning, IAP fluctuates markedly with respiration, abdominal muscle activity, and shear deformation of the visceral organs due to intra-abdominal fat depositions and changes in the filling status of the hollow organs (Loring et al., 1994; De Keulenaer et al., 2009; Wilson et al., 2010). Sheep have a digestive tract consisting of four stomachs: the rumen, the reticulum, the omasum, and the abomasum. The rumen is the first and largest digestive organ, occupying the entire left part of the abdomen and accounting for 50–60% of the abdominal volume (Medjekal and Ghadbane, 2021). The ruminal content is partitioned into three primary zones based on their specific gravity. Gas rises to fill the upper regions, grain and fluid-saturated roughage sink to the bottom, and newly arrived roughage floats in a middle layer. IAP measurements in goats have been shown to coincide with the fluid surface level within the rumen. Higher pressures were recorded on ventral positions in the abdomen, and were shown to be directly proportional to vertical distance to the fluid surface (Hartman, 1973).

Interestingly, pressures recorded at identical points on the two sides of the abdomen were comparable (Hartman, 1973). The striking effect of the circular (from ventral to dorsal) ruminal contractions mixing up the ruminal contents once or twice per minute makes the sheep an intriguing animal model in abdominal pressure gradient assessment (Ruckebusch and Tomov, 1973). While IVP measurements in our setting delivered comparable values between all sheep, inter-individual variability of measurements in the four quadrants was much higher. While all sheep were fasted for 16–18 h prior to surgery, individual filling levels of the rumen and different nutritional status are plausible explanations for the increased variance. In 2/6 sheep (sheep A and D), IAP measurements followed gravitational gradients described by Hartman (1973) (Dagal and Lam, 2009). Namely, increased ventral and cranial pressures and lowered dorsal and caudal pressures. Increased dorsal pressure was measured in 3/6 sheep (Sheep C, E, and F). Reticulo-ruminal hypomotility caused by general anesthesia subsequently leads to a suppression of gas eructation and the onset of bloat (Dunlop et al., 1997). Although an orogastric tube was placed in all sheep to avoid any increased accumulation of gas, obstruction or dislodgement of the tube might have caused ruminal bloat and increased dorsal pressure in these three sheep. Abdominal pressures in the ventilated sheep showed different curve forms than in the spontaneous breathing sheep, which needs to be considered in VP shunt development using sheep as an animal model.

## Limitations

The sheep is a particularly interesting animal model in which to investigate CSF dynamics and interdependencies with adjacent compartments, namely the intra-abdominal cavity and the vascular compartments. Its unique digestive system offers a genuine opportunity to link intra-abdominal pressure gradients with alterations in hemodynamics and CSF pressure fluctuations and vice versa.

However, one must bear in mind a few things regarding the transferability of the model to humans. Firstly, the anatomic dimensions of the ovine CSF space are small. The total CSF volume in sheep is estimated to be only 25 mL compared with 150 mL in humans, consequently affecting the turnover rate (Mollanji et al., 2001; Sakka et al., 2011). Special care must be taken to minimize CSF loss during catheterization, as volume normalization time will be increased. Furthermore, reduced CSF turnover rate due to the smaller ventricular system must be considered when performing CSF-specific diagnostics such as lumbar taps or infusion tests. In addition, according to Thiéry et al. (2009), the turnover rate of CSF in female sheep depends on the light-dark cycle and can therefore vary over the year. Since the experiments in this study were performed during the short-day period of winter, the CSF turnover rate in sheep used for the experiments is believed to be increased. This physiological phenomenon could have influenced the CSF dynamics studied by Podgoršak et al. (2022) but is assumed not to have impacted the results presented here. However, seasonal alterations in the physiology of sheep need to be considered when conducting translational research with this species.

Secondly, sheep do not have an internal jugular vein, which could translate into different venous-dependent influences on CSF dynamics compared with humans and other species.

Thirdly, general anesthesia and mechanical ventilation can significantly impact all physiological pressures and measurements. When keeping the animal in an optimized anesthetic plane still allowing autoregulatory mechanisms to function, sound knowledge of the interdependencies of the compartments can be gained. However, no statement on absolute pressure levels should be made under general anesthesia.

Lastly, CSF flow through current VP shunt valves is driven by differential pressure and is thus a function of the ICP and the IAP as well as hydrostatic posture-related pressure gradient variations. The hydrostatic pressure in the quadruped animal is not directly comparable to the upright standing human - a factor that must be considered when developing novel shunt systems using animal models.

## Conclusion

The acute sheep model presented here offers the possibility of a great variety of interventions, such as exhausted CSF volume changes, induction of acute intra-abdominal hypertension, and controlled hemodynamic manipulations, that would not be feasible in a conscious animal. We are convinced that an enhanced understanding of the physiological mechanism can be gained from this model to improve medical devices such as VP shunts. Furthermore, we believe that the model developed is suitable for further *in vivo* investigations and can also support the derivation of mathematical models for test benches.

## Data Availability Statement

The raw data supporting the conclusions of this article will be made available by the authors, without undue reservation.

## Ethics Statement

The animal study was reviewed and approved by the Committee for Experimental Animal Research (Cantonal Veterinary Office Zurich, Switzerland).

## Author Contributions

NT searched the literature and wrote the manuscript. MW, MS, and MA developed the trial concept and study design. AP, MS, NT, and MW were involved in data acquisition. AP was responsible for post-processing of the data. MO provided the neuroscience part and performed the neurosurgical operations. NT and SJ were assisting in the surgical procedure. MW and NT performed the vascular catheterization and surgical placement of abdominal pressure probes. NT, MW, and SJ were responsible for animal care and anesthesia. All authors revised the manuscript and read and approved the final version of the manuscript.

## Conflict of Interest

The authors declare that the research was conducted in the absence of any commercial or financial relationships that could be construed as a potential conflict of interest.

## Publisher’s Note

All claims expressed in this article are solely those of the authors and do not necessarily represent those of their affiliated organizations, or those of the publisher, the editors and the reviewers. Any product that may be evaluated in this article, or claim that may be made by its manufacturer, is not guaranteed or endorsed by the publisher.

## Abbreviations

CSF: cerebrospinal fluid
ICP: intracranial pressure
iNPH: idiopathic normal pressure hydrocephalus
VP: ventriculoperitoneal
VA: ventriculoatrial
CPP: cerebral perfusion pressure
IAP: intra-abdominal pressure
CVP: central venous pressure
MAP: mean arterial pressure
IVP: intravesical pressure
cABP: carotid arterial blood pressure
fABP: femoral arterial blood pressure
JVP: jugular venous pressure
ITP: intrathecal pressure
IAP_cr_: cranial intra-abdominal pressure
IAP_cd_: caudal intra-abdominal pressure
IAP_ventr_: ventral intra-abdominal pressure
IAP_ds_: dorsal intra-abdominal pressure
FFT: Fast Fourier Transform
mfABP: mean femoral arterial pressure
mcABP: mean carotid arterial pressures
ICH: intracranial hypertension
ABP: arterial blood pressure.
